# Metformin Protects against H_2_O_2_-Induced Cardiomyocyte Injury by Inhibiting the miR-1a-3p/GRP94 Pathway

**DOI:** 10.1016/j.omtn.2018.09.001

**Published:** 2018-09-06

**Authors:** Ying Zhang, Xue Liu, Lu Zhang, Xuelian Li, Zhongqiu Zhou, Lei Jiao, Yingchun Shao, Mengmeng Li, Bing Leng, Yuhong Zhou, Tianyi Liu, Qiushuang Liu, Hongli Shan, Zhimin Du

**Affiliations:** 1 Department of Pharmacology (State-Province Key Laboratories of Biomedicine-Pharmaceutics of China, Key Laboratory of Cardiovascular Research, Ministry of Education), College of Pharmacy, Harbin Medical University, Harbin, Heilongjiang 150081, P.R. China; 2 Institute of Clinical Pharmacy, The Second Affiliated Hospital of Harbin Medical University, Harbin, Heilongjiang 150081, P.R. China; 3 Pharmacy Intravenous Admixture Service, The First Affiliated Hospital of Harbin Medical University, Harbin, Heilongjiang 150001, P.R. China; 4 Department of Pharmaceutics, Dalian Children’s Hospital, Dalian, Liaoning 116001, P.R. China

**Keywords:** microRNA, ischemia-reperfusion injury, endoplasmic reticulum stress

## Abstract

Ischemia-reperfusion (I/R) injury is a major side effect of the reperfusion treatment of the ischemic heart. Few therapies are available for the effective prevention of this injury caused by the oxidative stress-induced cardiomyocyte apoptosis. Metformin was shown to have a potential cardiac protective effect and ability to reduce cardiac events, but the exact mechanism remains unclear. Here, we aimed to confirm and investigate the mechanisms underlying potential metformin activity against I/R injury in response to oxidative stress. We determined that the expression of miR-1a-3p was significantly increased in neonatal rat ventricular cells (NRVCs), which were exposed to H_2_O_2_*in vitro* and in the hearts of mice that underwent the I/R injury. MiR-1a-3p was shown to target the 3′ UTR of GRP94, which results in the accumulation of un- or misfolded proteins, leading to the endoplasmic reticulum (ER) stress. The obtained results demonstrated that C/EBP β directly induces the upregulation of miR-1a-3p by binding to its promoter. Furthermore, as a direct allosteric AMPK activator, metformin was shown to activate AMPK and significantly reduce C/EBP β and miR-1a-3p levels compared with those in the control group. In conclusion, metformin protects cardiomyocytes against H_2_O_2_ damage through the AMPK/C/EBP β/miR-1a-3p/GRP94 pathway, which indicates that metformin may be applied for the treatment of I/R injury.

## Introduction

Myocardial infarction (MI) remains a leading cause of mortality and morbidity worldwide.^1^, ^2^ Although thrombolysis and direct coronary intervention have saved millions of lives, myocardial injury followed by the restoration of blood flow remains a complication of arterial recanalization therapy,^3^ and only a few effective therapies are available for prevention of myocardial ischemia-reperfusion (I/R) injury. Therefore, the identification of more effective treatments for cardiac I/R injury is necessary. To date, several mechanisms underlying cardiac I/R injury have been proposed: oxidative stress,^4^ cellular calcium overload,^5^ and inflammatory response.^6^

Metformin is the first-line medication for the treatment of type 2 diabetes; moreover, it is reported to have cardiac protective effects and may help reduce cardiac events.^7^, ^8^ Long-term (12 weeks) metformin administration has shown to preserve cardiac function in a rat model of post-MI cardiac remodeling by reducing infarct size, improving left ventricular (LV) geometry, and through other mechanisms.^9^ Further research demonstrates that metformin protects the ischemic heart through the Akt-mediated inhibition of the opening of mitochondrial permeability transition pore (mPTP).^10^ However, metformin for the treatment of H_2_O_2_-induced oxidative stress has not been extensively elucidated.

MicroRNAs (miRNAs), a class of endogenous ∼22-nt-long non-coding RNAs, can induce post-transcriptional inhibition of gene expression by targeting the 3′ UTR of messenger RNAs harboring partially complementary sequences.^11^, ^12^ An increasing number of miRNAs are reported to regulate the development of cardiac pathological processes, including heart failure, myocardial fibrosis, atrial fibrillation, cardiac hypertrophy, MI, and others. For example, miR-328 induces atrial fibrillation by promoting atrial electrical remodeling,^13^ and miR-1 regulates cardiac arrhythmogenic potential by targeting relevant ion-channel-encoding genes KCNJ2 and gap junction protein alpha 1 (GJA1) in cardiac ischemic injuries.^14^ However, the possible roles of miR-1 in cardiac I/R injury have not been investigated previously.

In this study, we exposed neonatal rat ventricular cells (NRVCs) to H_2_O_2_, in order to imitate oxidative stress followed by cardiac I/R injury and to identify the regulatory mechanisms underlying this process. Additionally, we aimed to determine the effects of metformin on the cardiac I/R injury and explore the mechanisms involving miRNAs.

## Results

### Metformin Attenuates H_2_O_2_-Induced Cardiomyocyte Injury through the Inhibition of miR-1a-3p Expression and Promotion of GRP94 Expression

**Figure 1:**
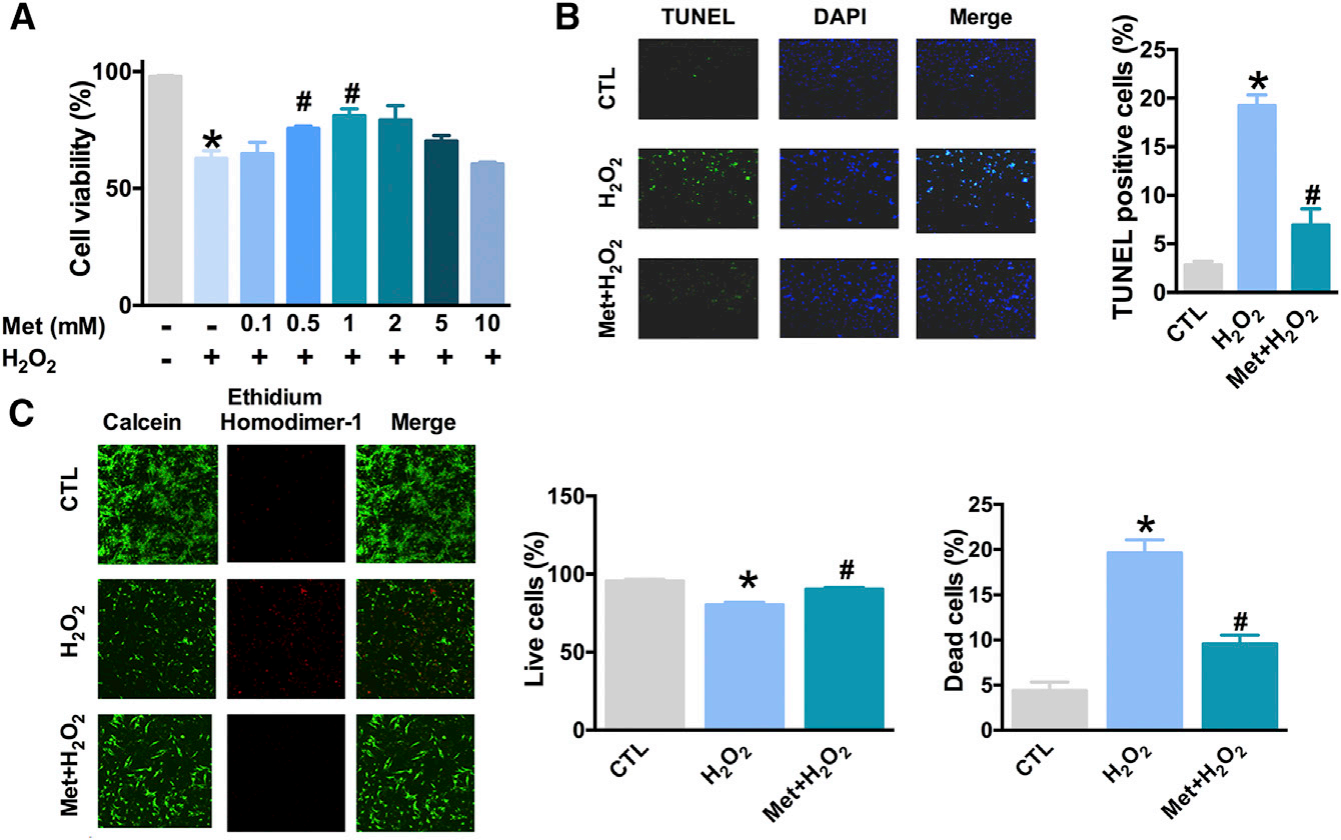
Metformin Attenuates H_2_O_2_-Induced Cardiomyocyte Apoptosis (A) Cell viability was determined with MTT assay. n = 4; *p < 0.05 versus CTL group (without treatment by Met or H_2_O_2_); ^#^p < 0.05 versus H_2_O_2_-only-treated group. (B) Left, TUNEL assay results. Right, relative percentage of TUNEL-positive cardiomyocytes. n = 5; *p < 0.05 versus CTL group; ^#^p < 0.05 versus H_2_O_2_-only-treated group. (C) Left, LIVE/DEAD assay results. Middle, relative percentage of live cells. Right, relative percentage of dead cells. n = 6; *p < 0.05 versus CTL group, ^#^p < 0.05 versus H_2_O_2_-only-treated group. All of the data are presented as the mean ± SEM.

To determine whether metformin could protect primary cardiomyocytes against H_2_O_2_-induced cell injury *in vitro*, we incubated the cardiomyocytes with metformin (0.1, 0.5, 1, 2, 5, or 10 mM) for 30 min before the addition of H_2_O_2_ and determined cell viability with the MTT assay. The viability of cardiomyocytes was shown to decrease following the treatment with 100 μM H_2_O_2_ for 48 hr, and it increased when the cardiomyocytes were pretreated with 0.5 mM or 1 mM metformin, compared with that in the samples treated with H_2_O_2_ only (Figure 1A). Additionally, TUNEL assay results showed that 1 mM metformin pretreatment could rescue H_2_O_2_-induced DNA damage (Figure 1B). Moreover, 1 mM metformin treatment induced the survival of cardiomyocytes and decreased the death of these cells (Figure 1C).

**Figure 2:**
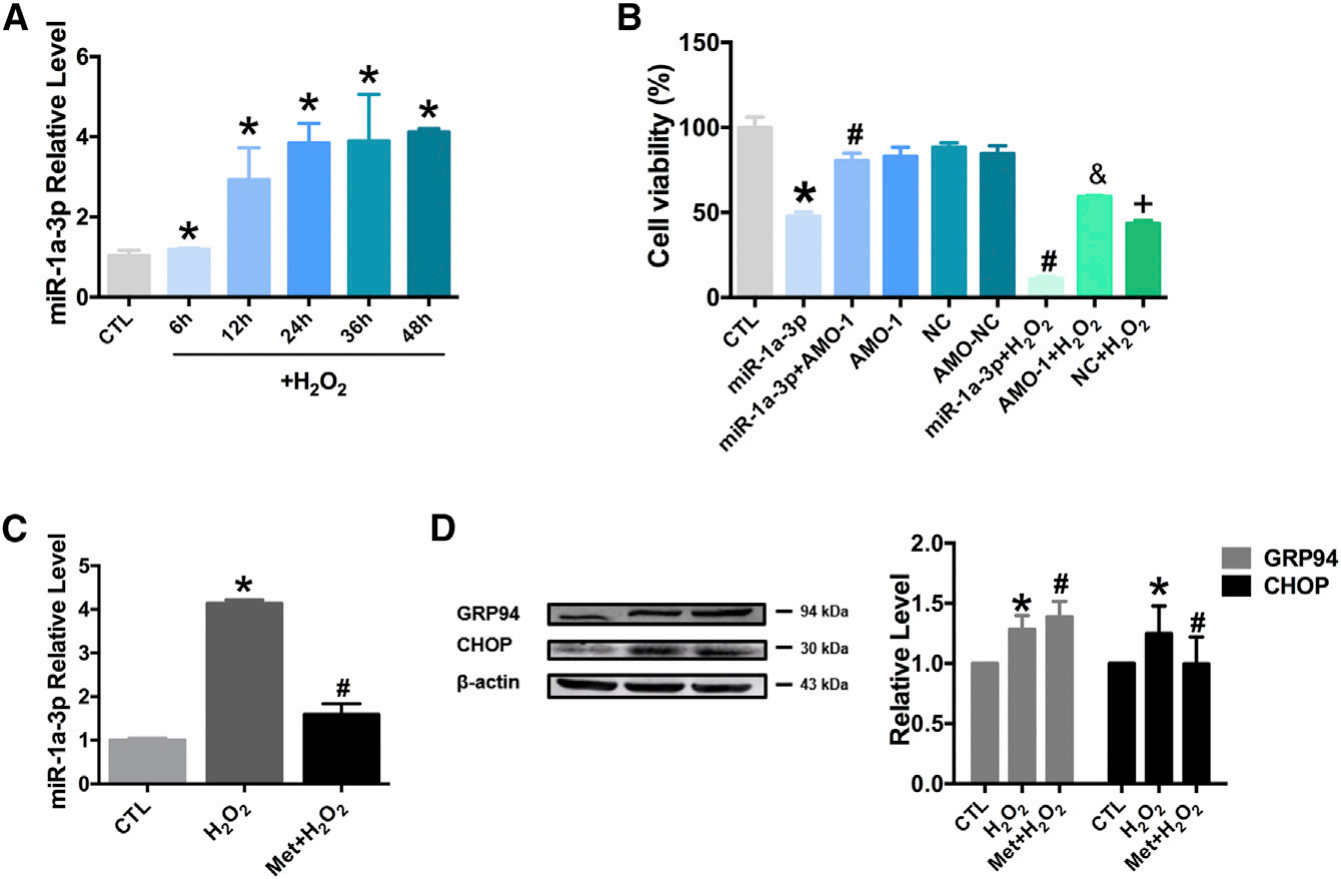
Metformin Inhibits miR-1a-3p Expression and Promotes GRP94 Expression (A) Effect of H_2_O_2_ on the expression of miR-1a-3p at different time points. n = 3; *p < 0.05 versus CTL group. (B) Effect of miR-1a-3p on NRVC viability after H_2_O_2_ treatment. n = 5; *p < 0.05 versus CTL group, ^#^p < 0.05 versus miR-1a-3p group, ^&^p < 0.05 versus AMO-1 group, ^+^p < 0.05 versus NC group. (C) Effect of Metformin (1 mM) pretreatment before H_2_O_2_ on elevation of miR-1a-3p. n = 3; *p < 0.05 versus CTL group, ^#^p < 0.05 versus H_2_O_2_-only-treated group. (D) Effect of Metformin (1 mM) on GRP94 and CHOP expression. n = 4; *p < 0.05 versus CTL group, ^#^p < 0.05 versus H_2_O_2_-only-treated group. All of the data are presented as the mean ± SEM.

The expression of miR-1a-3p under oxidative stress was evaluated by real-time PCR in cultured NRVCs that are exposed to 100 μM H_2_O_2_ at different time points. MiR-1a-3p levels showed a time-dependent increase (Figure 2A). To evaluate miR-1a-3p-induced cardiomyocyte injury, cardiomyocyte viability was determined following the transfection with miR-1a-3p or AMO-1. MiR-1a-3p transfection was shown to induce a decrease in cardiomyocyte viability and exacerbate H_2_O_2_-induced cardiomyocyte injury. However, cardiomyocyte viability was shown to recover when the cells were transfected with AMO-1 before H_2_O_2_ treatment (Figure 2B). MiR-1a-3p levels were shown to be 4.14-fold higher in the cardiomyocytes treated with H_2_O_2_ for 48 hr compared with those in the untreated cells, but these levels were shown to decrease in the cardiomyocytes pretreated with 1 mM of metformin for 0.5 hr (Figure 2C). Additionally, metformin significantly induced the increased expression of GRP94 (involved in the disposal of misfolded protein), compared with that in the control group, and decreased the expression of CHOP, which induces apoptosis (Figure 2D).

### MiR-1a-3p Aggravates ER Stress by Inhibiting GRP94 Expression

**Figure 3:**
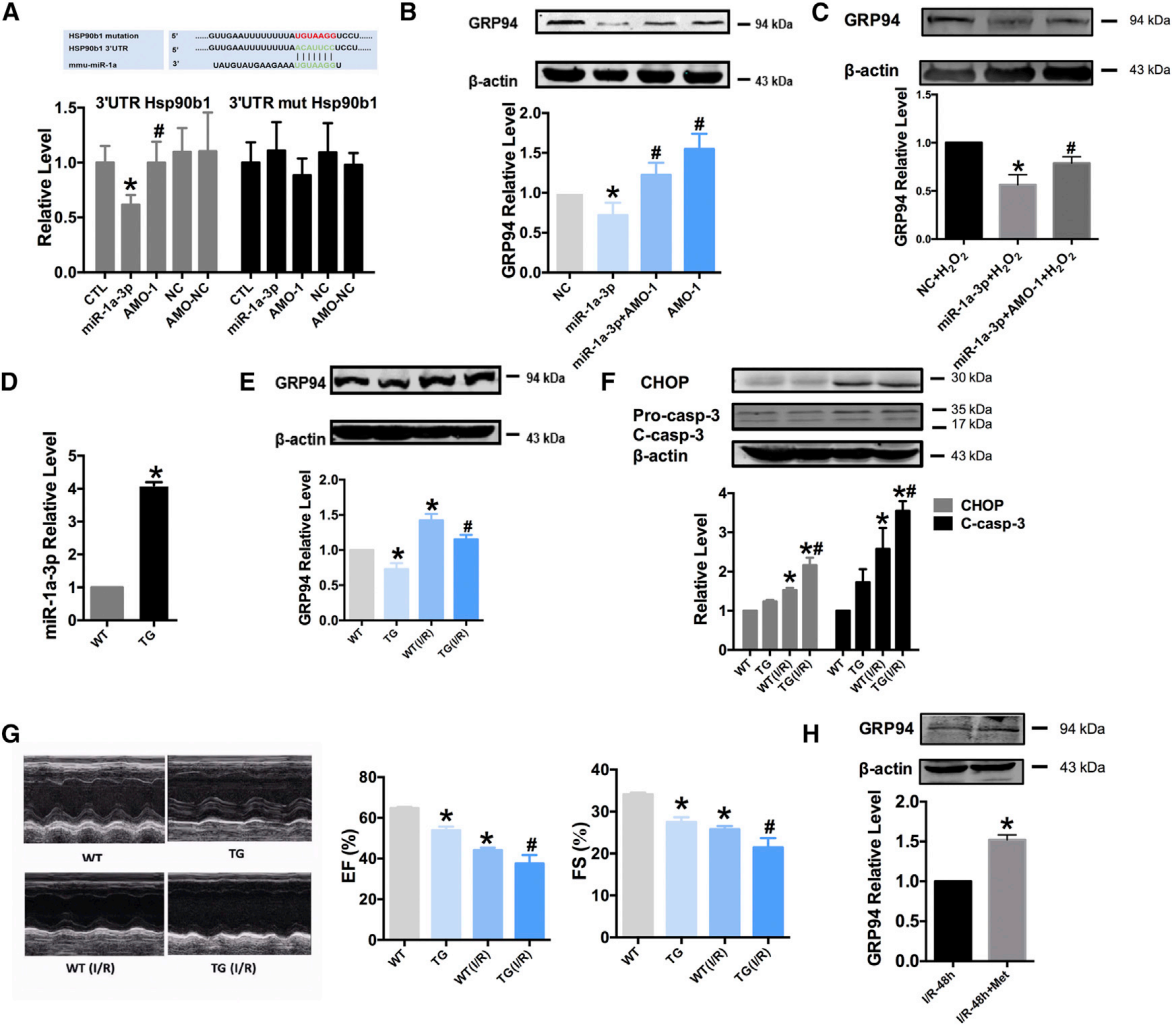
MiR-1a-3p Inhibits GRP94 Expression by Binding to 3′ UTR Binding Sites of Hsp90b1 (A) Complementarity between miR-1a-3p seed sequence and the 3′ UTR (position 172–178) of Hsp90b1 predicted by Targetscan. Highlight, mutations introduced into the gene sequence (upper). Luciferase reporter gene assay, demonstrating the inhibition of GRP94 (Hsp90b1) expression by miR-1a-3p in HEK293T cells (lower). n = 3; *p < 0.05 versus CTL group, ^#^p < 0.05 versus miR-1a-3p group. (B) The expression of GRP94 was significantly reduced by miR-1a-3p in NRVCs. n = 6; *p < 0.05 versus NC group, ^#^p < 0.05 versus miR-1a-3p group. (C) Effect of miR-1a-3p on GRP94 levels after H_2_O_2_ treatment. n = 3; *p < 0.05 versus NC + H_2_O_2_ group, ^#^p < 0.05 versus miR-1a-3p + H_2_O_2_ group. (D) The expression of miR-1a-3p in WT or TG mice. n = 5; *p < 0.05 versus WT group. (E) The expression of GRP94 in the WT, TG, WT (I/R), and TG (I/R) mice. n = 5; *p < 0.05 versus WT group, ^#^p < 0.05 versus WT (I/R) group. (F) The expression of CHOP and C-Caspase-3 in the WT, TG, WT (I/R), and TG (I/R) mice. n = 4; *p < 0.05 versus WT group, ^#^p < 0.05 versus WT (I/R) group. (G) Representative M-mode echocardiography images and echocardiographic analysis of volumes and left ventricular ejection fraction (EF, n = 5–19) and left ventricular fractional shortening (FS, n = 8–23); *p < 0.05 versus WT group, ^#^p < 0.05 versus WT (I/R) group. (H) The expression of GRP94 in mice with I (50 min)/R (48 hr) with or without metformin administration. n = 3; *p < 0.05 versus WT (I/R) group. All of the data are presented as the mean ± SEM.

Furthermore, we performed bioinformatic analysis with TargetScan 6.0 and miRanda. We focused on GRP94 (Hsp90b1), since it was shown to have critically conserved sites and total context score, as a possible target. To confirm the direct interaction between miR-1a-3p and the 3′ UTR of Hsp90b1, we cloned the wild-type and mutant 3′ UTRs of Hsp90b1, containing miR-1a-3p binding sites, into the luciferase reporter vector. Afterward, we co-transfected the constructs with miR-1a-3p, AMO-1, negative control (NC), and AMO-NC into HEK293T cells and assessed the effects of miR-1a-3p on luciferase activity. As shown in Figure 3A, relative luciferase activity was reduced when the cells were co-transfected with miR-1a-3p and the wild-type Hsp90b1 constructs, whereas the mutation of the binding sites abolished miR-1a-3p effects. For further confirmation, miR-1a-3p and AMO-1 were transfected using Lipofectamine 2000 into the cultured NRVCs. We determined that miR-1a-3p could inhibit the expression of GRP94, compared with that in the NC group, while the co-transfection with AMO-1 prevents this inhibition with or without H_2_O_2_ (Figures 3B and 3C). We confirmed this regulatory relationship in a cardiac-specific miR-1a-3p transgenic mouse (TG) by inserting the precursor sequence of miR-1a-3p carrying the promoter of the cardiac-specific α myosin heavy chain (αMHC) into the mouse genomes. Heterozygous litters were shown to have a significant increase in miR-1a-3p levels (4.10-fold), compared with those in the wild-type (WT) mice (Figure 3D). Therefore, ER stress was shown to underlie MI or I/R injury. GRP94 is an ER chaperone protein, involved in the alleviation of ER stress and the maintenance of ER function.^15^ As shown in Figure 3E, ER stress was induced by the I/R injury in both WT and TG mice, while the expression of GRP94 was shown to be higher in TG (I/R) mice than in the TG mice. Additionally, we determined a considerable increase in CHOP expression in the TG (I/R) group, compared with that in the WT (I/R) group (Figure 3F). ER stress-induced apoptosis was shown to be activated after mice suffered the I/R injury, which was associated with the increase in the expression of cleaved Caspase-3 (C-Casp-3), and the expression of this molecule was determined to be significantly higher in the TG (I/R) mice than in the WT (I/R) mice (Figure 3F). TG mice were shown to have more severe cardiac injuries after I/R injury, with ejection fraction (EF) and fractional shortening (FS) values decreased in TG (I/R) mice compared with those in the WT (I/R) mice (Figure 3G). After administration of metformin (200 mg/kg/day for 2 weeks), the expression of GRP94 was upregulated in the WT (I/R) mice (Figure 3H).

### Metformin Induces AMPK Phosphorylation and Inhibits C/EBP β Expression

**Figure 4:**
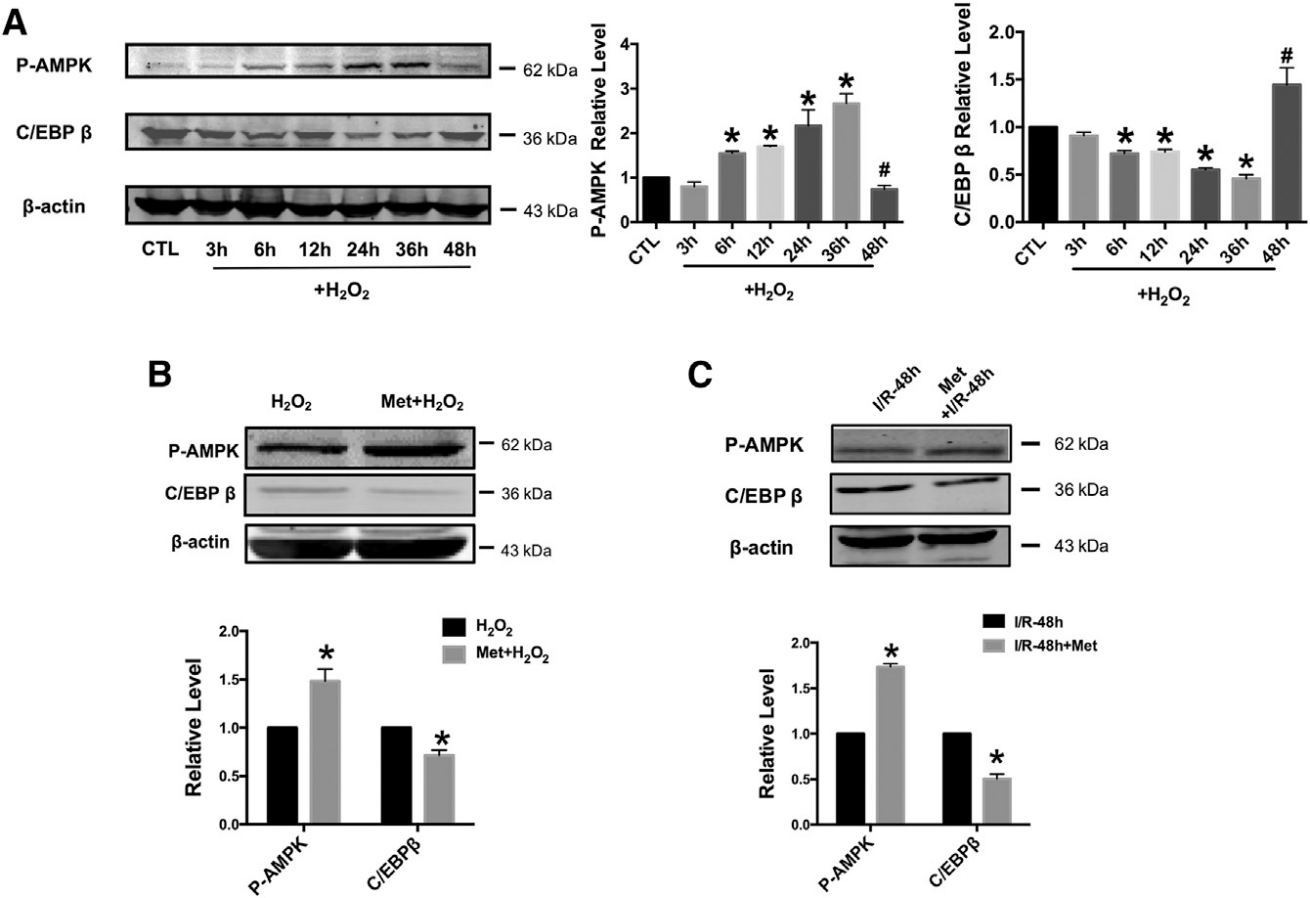
Metformin Induces AMPK Phosphorylation and Inhibits C/EBP β Expression (A) The expression of AMPK phosphorylation and C/EBP β after H_2_O_2_ treatment at different time points. n = 3; *p < 0.05 versus CTL group; ^#^p < 0.05 versus H_2_O_2_-treated for 36 hr. (B) The expression of AMPK phosphorylation and C/EBP β after H_2_O_2_ treatment and metformin pretreatment. n = 4–6; *p < 0.05 versus H_2_O_2_-treated only group. (C) The expression of AMPK phosphorylation and C/EBP β in mice with I (50 min)/R (48 hr) with or without metformin administration. n = 3; *p < 0.05 versus WT (I/R) group. All of the data are presented as the mean ± SEM.

Furthermore, we determined AMPK phosphorylation levels at Thr172. AMPK phosphorylation was shown to increase in a time-dependent manner within 36 hr treatment of H_2_O_2_, but it decreased sharply at 48 hr after the imitation of H_2_O_2_ treatment (Figure 4A). In contrast to this, the expression of CCAAT/enhancer-binding protein beta (C/EBP β) was shown to increase at 48 hr after the initiation of H_2_O_2_ treatment. As an AMPK activator, metformin increased the phosphorylation of AMPK by 1.5-fold in the H_2_O_2_-treated cardiomyocytes, compared with that in the control samples (Figure 4B). However, the expression of C/EBP β was decreased by 20% after metformin pretreatment, compared with that in the control samples. Similarly, the phosphorylation level of AMPK was improved and the expression of C/EBP β was decreased in the I/R mice after administration of metformin for 2 weeks (Figure 4C).

### Role of C/EBP β in the Upregulation of miR-1a-3p Expression

**Figure 5:**
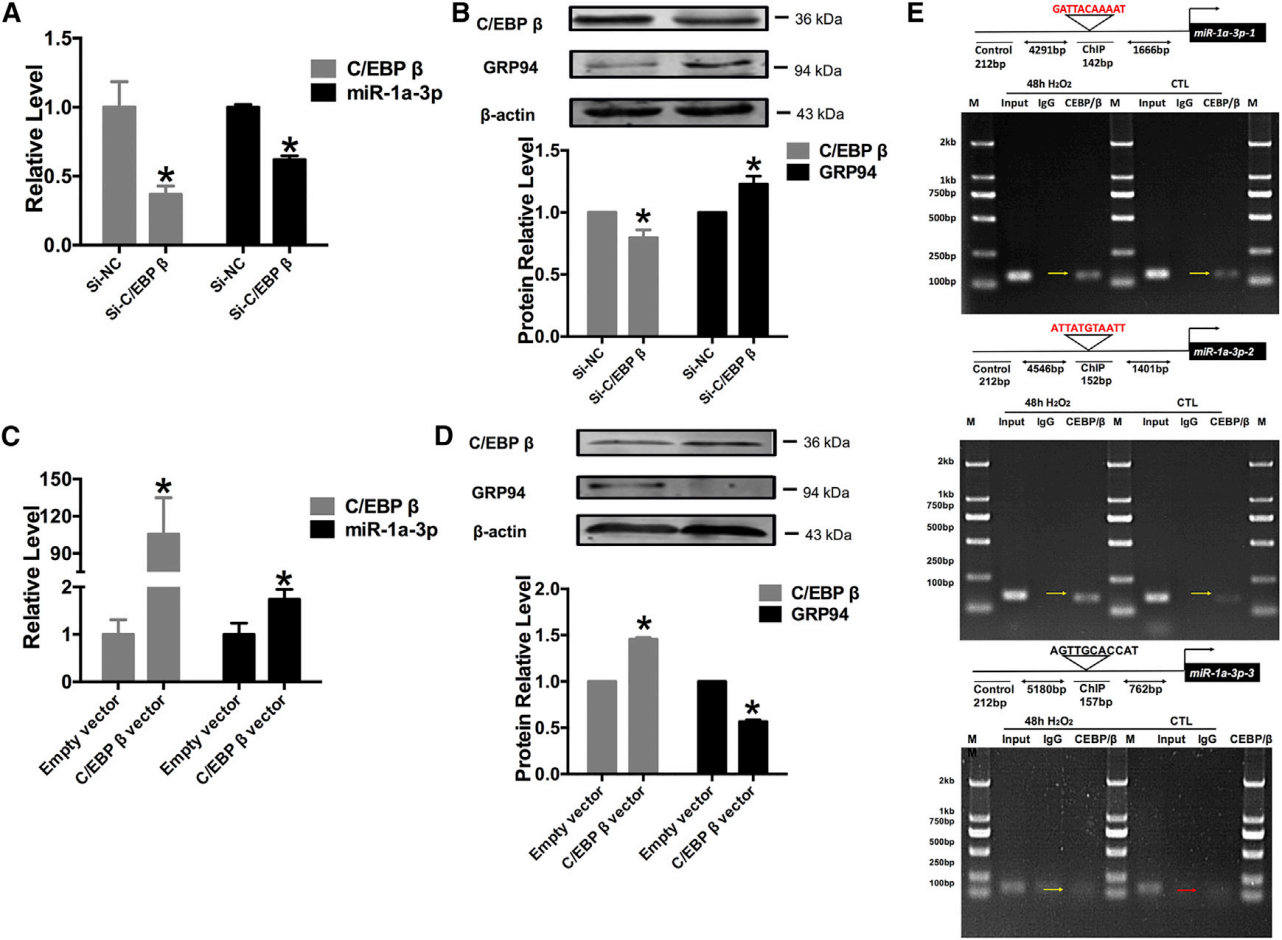
Role of CCAAT/Enhancer-Binding Protein Beta in Upregulation of miR-1a-3p (A) Both CCAAT/enhancer-binding protein beta (C/EBP β) and miR-1a-3p were decreased after silencing C/EBP β on RNA level. n = 9; *p < 0.05 versus Si-NC group. (B) The expression of C/EBP β and GRP94 after silencing C/EBP β. n = 6; *p < 0.05 versus Si-NC group. (C) Both C/EBP β and miR-1a-3p were increased after overexpression of C/EBP β on RNA level. n = 4; *p < 0.05 versus Empty vector group. (D) The expression of C/EBP β and GRP94 were increased after overexpression of C/EBP β. n = 4; *p < 0.05 versus Empty vector group. (E) Schematic illustration of the miR-1a-3p upstream promoter containing C/EBP β-binding sites. ChIP analyses revealed that H_2_O_2_ treatment could promote C/EBP β binding to the promoter of pre-miR-1a-3p, leading to miR-1a-3p overexpression in cardiomyocytes. All of the data are presented as the mean ± SEM.

The inverse relationship between C/EBP β and GRP94 expression was determined using the transient knockdown or overexpression C/EBP β in NRVCs *in vitro* (Figures 5A and 5B). C/EBP β and miR-1a-3p were shown to be significantly downregulated following the transient knockdown of this gene using small interfering RNA (siRNA), which was accompanied by a significant increase in GRP94 expression (Figure 5B). In contrast to this, C/EBP β overexpression led to the increase in C/EBP β protein expression and miR-1a-3p levels in NRVCs *in vitro* (Figure 5C), further inducing a reduction in GRP94 expression, compared with the control samples (Figure 5D). Using computational analyses, we determined three C/EBP β binding sites in the pre-miR-1a-3p promoter (−2,237 to 87 bp): (−1,516) GATTACAAAAT (−1,506), (−1,252) AATTACATAAT (−1,242), and (−689) AGTTGCACCAT (−679). Chromatin immunoprecipitation (ChIP) analyses revealed that C/EBP β could specifically bind to its two binding sites (−1,515 to −1,506 and −1,252 to −1,242) located in the promoter of pre-miR-1a-3p, providing convincing evidence that C/EBP β could directly regulate miR-1a-3p expression. Furthermore, H_2_O_2_ treatment was shown to promote C/EBP β binding to the promoter of pre-miR-1a-3p, leading to miR-1a-3p overexpression (Figure 5E).

## Discussion

**Figure 6:**
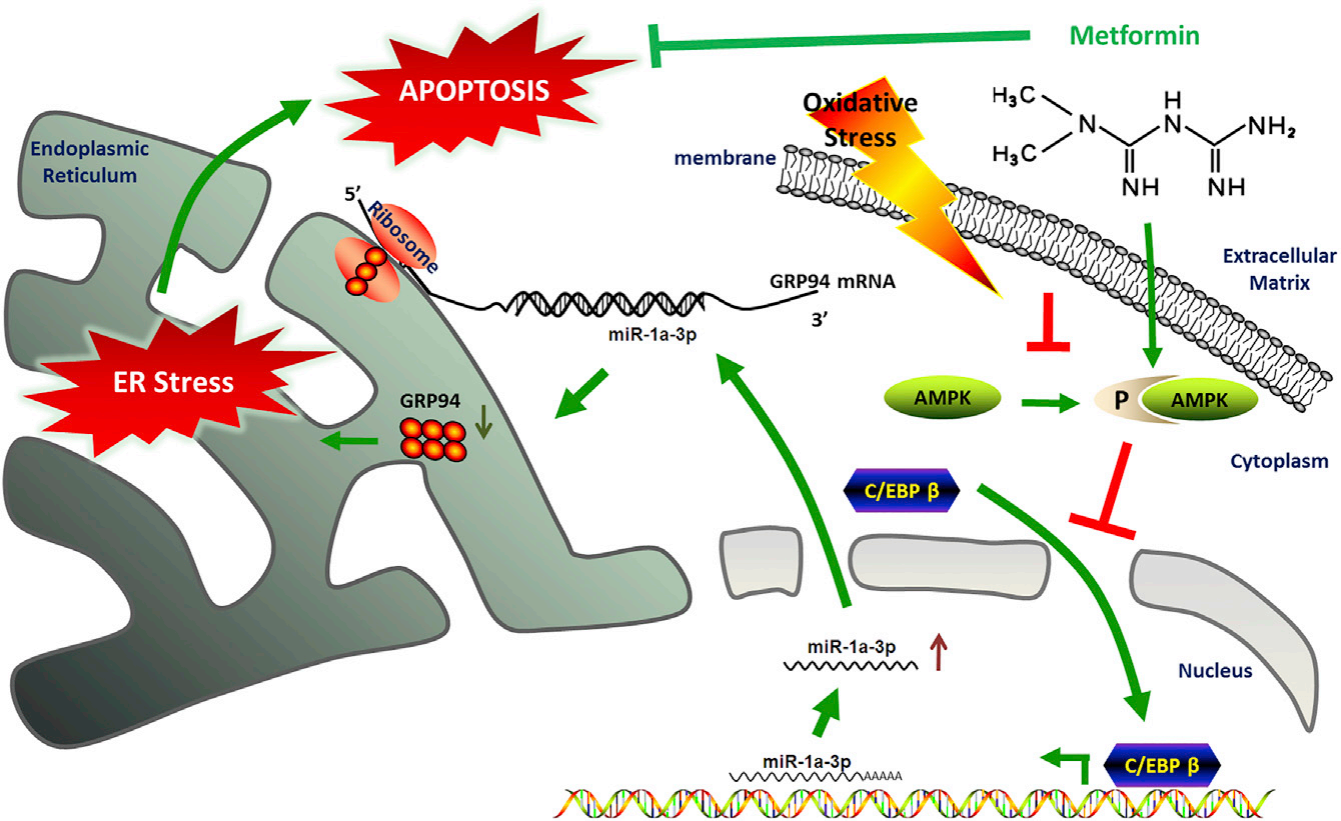
Metformin Improves Cardiomyocytes through the AMPK/C/EBP β/miR-1a-3p/GRP94 Pathway Schematic cartoon of the mechanism of metformin’s effect on H_2_O_2_-induced cardiomyocyte injury. Metformin was shown to promote AMPK phosphorylation and suppress C/EBP β expression, and C/EBP β knockdown may finally lead to a decrease in miR-1a-3p expression and an increase in GRP94 (the target gene of miR-1a-3p). Above all, it may protect cardiomyocytes against apoptosis.

For the first time, we demonstrated that metformin could improve cardiomyocyte viability after H_2_O_2_ treatment and showed that the C/EBP β/miR-1a-3p/GRP94 signaling pathway was at least partially responsible for these protective effects. These findings indicated the potential of metformin for the prevention and treatment of cardiac I/R injury (Figure 6).

High incidence of cardiac I/R injury after the restoration of blood flow in the infarcted myocardium leads to the severe impairment of patients’ health. Oxidative stress was reported to be crucial for the development of the I/R injury.^16^ However, relatively little is known about the exact mechanisms underlying this process. As commonly found reactive oxygen species, H_2_O_2_ (<600 μM) was generally used in the previous studies to induce the changes mimicking I/R injury *in vitro*.^17^ Here, we used this approach, as well for the treatment of NRVCs.

Although metformin is the first-line medication used for the control of blood glucose levels, it was reported to have protective effects against cardiovascular injury as well. Wang et al.^18^ reported that metformin has beneficial effects on H_2_O_2_-treated NRVCs. The results of our study showed that H_2_O_2_ treatment induces a significant decrease in cardiomyocyte viability, compared with that of the control cells, while the incubation with 0.5 mM and 1.0 mM metformin protected cardiomyocytes from the harmful effects of H_2_O_2_ treatment. Additionally, the protective effects of metformin were further confirmed by the observed reduction in cardiomyocyte apoptosis and increase in their viability.

As important regulators, miRNAs were shown to regulate some pathological processes in heart. Among these, miR-1 is considered a muscle-specific miRNA,^19^ and it was reported to slow down the conduction and depolarize cytoplasmic membrane through post-transcriptional inhibition of *KCNJ2* (encoding for K^+^ channel subunit Kir2.1) and *GJA1* (connexin 43) expression.^14^ Furthermore, it was shown that the inhibition of CDK6 and activation of retinoblastoma (Rb) pathway contribute to the attenuation of miR-1a-3p effects on provoking cardiomyocyte hypertrophy.^20^ Additionally, miR-1a-3p was found to induce heart dysfunction by inhibiting the expression of SOD1, GCLC, and G6PD, likely contributing to the increase in reactive oxygen species (ROS) levels.^21^ Here, we demonstrated that metformin inhibited the expression of miR-1a-3p in response to oxidative stress, indicating a novel mechanism of metformin activity in the protection of cardiomyocytes against oxidative stress.

miRNAs are emerging as central regulators in many cardiac pathological processes. As a muscle-specific miRNA, miR-1a-3p is believed to be the key regulator of cardiac diseases. This molecule exacerbates arrhythmogenesis^14^ and is involved in nitric oxide-induced apoptosis,^22^ and aggravated cardiac oxidative stress.^21^ In this study, we showed that miR-1a-3p induced a significant reduction in GRP94 expression by binding to the 3′ UTR of this gene, and the overexpression of miR-1a-3p inhibited GRP94 expression in NRVCs. Additionally, we determined that mice overexpressing miR-1a-3p in hearts had low levels of GRP94. This ER chaperone, could bind to misfolded proteins and unassembled complexes to stabilize the ER environment and protect against calcium dyshomeostasis and cell death.^23^, ^24^

Some compounds such as curcumin can induce a delayed cytoprotection against oxidative stress in myogenic cells by increasing GRP94 protein levels, which regulates calcium homeostasis.^23^ Our results showed that metformin can protect cells against H_2_O_2_-induced injury by decreasing miR-1a-3p levels and increasing GRP94 expression levels. In the cardiovascular system, metformin can activate AMPK,^25^ which regulates energy level, stimulates fatty acid oxidation,^26^, ^27^ accelerates glycolysis,^28^ and has other effects. C/EBP β, but not p-CREB, level was shown to be decreased by the AMPK activator AICAR or constitutively activated AMPK, while dominant-negative inhibitor of AMPK led to an increase in C/EBP β expression.^29^ Metformin, a direct allosteric AMPK activator,^30^ was shown to activate AMPK and suppress C/EBP β expression significantly, compared with that in the controls. Our further analyses demonstrated that C/EBP β knockdown may lead to a decrease in miR-1a-3p expression and an increase in GRP94 expression, and that it may protect cardiomyocytes against apoptosis. C/EBP β overexpression was shown to lead to a decrease in cardiomyocyte viability by increasing miR-1a-3p expression. Additionally, we experimentally showed that C/EBP β promoted miR-1a-3p expression directly.

In summary, we found that metformin can protect cardiomyocytes against H_2_O_2_-induced injury by modulating the AMPK/C/EBP β/miR-1a-3p/GRP94 pathway, which suggests a potentially important anti-oxidative stress effects of metformin during I/R.

## Materials and Methods

### Antibodies and Reagents

Metformin was purchased from Melone Pharmaceutical (Dalian, China). Anti-GRP94 and anti-C/EBP β antibodies were obtained from Sangon Biotech (Shanghai, China). Anti-CHOP and anti-Caspase-3 antibodies were purchased from Santa Cruz Biotechnology (Santa Cruz, CA, USA), and anti-phosphorylated (p)-AMPKα was from Cell Signaling Technology (Beverly, MA, USA). Anti-β-actin was obtained from ZSGB-BIO (Beijing, China).

### Animals

Neonatal (1- to 3-day-old) Sprague-Dawley (SD) rats and adult male C57BL/6 mice (25–30 g) were provided by the Experimental Animal Center of the Second Affiliated Hospital of Harbin Medical University (Harbin, China). Transgenic mice with the cardiac-specific overexpression of miR-1 were kindly provided by Professor Xu Gao (Department of Biochemistry and Molecular Biology, Harbin Medical University). All animal procedures were approved by the Institutional Animal Care and Use Committee of the Harbin Medical University. All procedures conformed to the Guide for the Care and Use of Laboratory Animals published by the US NIH.

### Cardiac I/R Injury and Echocardiographic Measurements

Metformin (200 mg/kg/day, intragastric administration) was used to treat male C57BL/6 mice for 2 weeks, then the mice were anaesthetized with sodium pentobarbital (50 mg·kg^−1^, intraperitoneally [i.p.]), and their respiration was controlled by a small ventilator. The mice were subjected to 50 min occlusion of the left anterior descending (LAD) coronary artery, followed by 48 hr of reperfusion. LV functional parameters, such as the LV systolic diameter (LVSd) and LV diastolic diameter (LVDd), were evaluated using transthoracic echocardiography (VisualSonics Vevo 2100, Toronto, Canada). LV EF and FS were calculated from M-mode recordings.

### Isolation of Primary NRVCs and Treatment

The procedures used for NRVC culturing have been described previously in detail.^31^ Cultured primary NRVCs were exposed to 1 mM metformin for 30 min and treated with 100 μM H_2_O_2_ afterward (Sigma-Aldrich, St. Louis, MO, USA) for 48 hr.

### TUNEL Assay

Cardiomyocytes were fixed with 4% paraformaldehyde and permeabilized with 0.1% Triton X-100. Cardiomyocyte apoptosis was detected by the *In Situ* Cell Death Detection Kit (Roche, Mannheim, Germany). The treated samples were observed under a fluorescence microscope (Olympus, Tokyo, Japan).

### Cell Viability Assessment Using MTT Assay

Cell viability was examined by using MTT [3-(4,5-dimethylthiazol-2-yl)-2,5-diphenyl-2 H-tetrazolium bromide] assay. NRVCs were plated into 96-well plates and incubated with 20 μL of MTT (0.5 mg/mL) for 4 hr. After removing the media gently, 200 μL of DMSO were added into each well to dissolve formazan. After shaking the samples for 10 min, the absorbances were measured at 570 nm by Infinite m200pro microplate spectrophotometer (Tecan, Salzburg, Austria).

### Cell Staining

Live and dead cells were detected by The LIVE/DEAD Viability/Cytotoxicity Assay Kit (Invitrogen, Shanghai, China) according to the manufacturer’s instructions.

### Transfection of Cells with miRNAs and siRNAs

MiR-1a-3p (5′-UGGAAUGUAAAGAAGUGUGUAU-3′, 5′-AUACACACUUCUUUACAUUCCA-3′), miR-1a-3p inhibitor (AMO-1, 5′- mAmUmAmCmAmCmAmCmUmUmCmUmUmUmAmCmAmUmUmCmCmA-3′, m, 2′-Ome), NC (5′-UUUGUACUACACAAAAGUACUG-3′, 5′-CAGUACUUUUGUGUAGUACAAA-3′), and NC inhibitor (5′-mCmAmGmUmAmCmUmUmUmUmGmUmGmUmAmGmUmAmCmAmAmA-3′, m, 2′-Ome) molecules were purchased from Ribobio (Guangzhou, China). C/EBP β siRNA (5′-AGCCGUCCGACUACGGUUATTUAACCGUAGUCGGACGGCUTT-3′) and their NC (si-NC) were purchased from Invitrogen (Shanghai, China). They were transfected into NRVCs with opti-MEMI serum-free culture medium (Gibco, New York, USA) and Lipofectamine 2000 (Invitrogen, Carlsbad, CA, USA) according to the manufacturer’s instruction.

### Western Blot

Total proteins extracted from NRVCs or mouse LV tissue samples were separated using SDS-PAGE (10%–15%) and transferred to nitrocellulose membranes (Life Sciences, Überlingen, Germany). After blocking the membranes with 5% non-fat milk, the samples were incubated with the primary antibodies against GRP94, p-AMPKα, C/EBP β, CHOP, Caspase-3, and β-actin at 4°C overnight. Afterward, membranes were washed with PBSe with Tween 20 (PBST) and incubated with the secondary antibody (Alexa Fluor, Molecular Probes, Eugene, OR, USA) for 50 min in dark. The obtained bands were quantified using Odyssey v1.2 software (LI-COR Biosciences, Lincoln, NB, USA). The results were normalized to the levels of β-actin.

### Real-Time PCR

Total RNA extraction from NRVCs or mouse LV tissue samples were performed as described previously.^14^ Real-time qPCR was performed using thermocycler ABI 7500 fast real-time PCR system (Applied Biosystems, Carlsbad, CA, USA). Rno-miR-1a-3p primer sequences were as follows:

RT, 5′-GTCGTATCCAGTGCGTGTCGTGGAGTCGGCAATTGCACTGGATACGACTACACAC-3′;
Forward, 5′-GGGGTGGAATGTAAAGAA-3′;
Reverse, 5′-TGCGTGTCGTGGAGTC-3′.

The primer sequence of mmu-miR-1a-3p was as follows:

RT, 5′-GTCGTATCCAGTGCGTGTCGTGGAGTCGGCAATTGCACTGGATACGACATACATAC-3′;
Forward, 5′-GGCGTGGAATGTAAAGAA-3′;
Reverse, 5′-CGGCAATTGCACTGGATA-3′.

The primer sequence of U6 was as follows:

RT, 5′-CGCTTCACGAATTTGCGTGTCAT-3′;
Forward, 5′-GCTTCGGCAGCACATATACTAAAAT-3′;
Reverse, 5′-CGCTTCACGAATTTGCGTGTCAT-3′.

The obtained results were normalized to the levels of the endogenous control (U6).

### Dual Luciferase Reporter Assay

HEK293T cells were transfected with 20 μmol/L miR-1a-3p, AMO-1, NC, or NC inhibitor (AMO-NC) together with 0.5 μg psi-CHECK2 target DNA. Luciferase activity was determined using Dual Luciferase Reporter Assay Kit (Promega, Madison, WI, USA) on luminometer (Promega, Madison, WI, USA) 48 hr after the transfection.

### ChIP

Cardiomyocytes were fixed in 1% mixing formaldehyde (Sigma-Aldrich, St. Louis, MO, USA) for 10 min to crosslink nucleoprotein complexes. Samples were then scraped in PBS with the protease inhibitor cocktail. Cardiomyocytes were lysed and transferred to lysis buffer. The sheared DNA complex was immunoprecipitated by incubating the lysates with 9 μg of anti-C/EBP β (Abcam, Cambridge, MA, USA) or immunoglobulin G (IgG) antibodies overnight. Crosslinks were shifted at 65°C for 40 min while stirring in 150 μL of ChIP elution buffer and 3 μL of Proteinase K after washing. DNA was separated by QIAquick PCR Purification Kit (Thermo Fisher Scientific, CA, USA). MiR-1a-3p promoter was amplified using PCR reaction. C/EBP β binding site was determined with miR-1a-3p-specific primers:

Forward1, 5′-GGCACAGGAACAGGGTGAATG-3′;
Reverse1, 5′-GAAATGATTCACAAGAGGGG-3′;
Forward2, 5′-ACCAATGTTGATTCAATTTTAG-3′;
Reverse2, 5′-CCTTTTAAGATAAATTTGAAAG-3′;
Forward3, 5′-GATCTGCATGAAGGTGTATGGC-3′;
Reverse3, 5′-CTTGAGAAGCTTTGAGATTTG-3′.

The obtained PCR products were analyzed using 1% agarose gel electrophoresis.

### Statistical Analysis

All data are reported as the mean ± SEM. Statistical analysis was used by one-way ANOVA followed by Dunnett’s t test. p < 0.05 was considered statistically significant.

## Author Contributions

H.S. and Z.D. designed and performed the research and supervised all aspects of the study and analysis. Y. Zhang, X.L., L.Z., and X.L. performed the study and analyzed the data. Z.Z., L.J., Y.S., M.L., B.L., Y. Zhou, T.L., and Q.L. assisted in this study. Y. Zhang, X.L., H.S., and Z.D. finalized the manuscript. Y. Zhang and L.Z. supervised the research.

## Conflicts of Interest

The authors declare no conflict of interest.
